# What accounts for individual differences in susceptibility to the McGurk effect?

**DOI:** 10.1371/journal.pone.0207160

**Published:** 2018-11-12

**Authors:** Violet A. Brown, Maryam Hedayati, Annie Zanger, Sasha Mayn, Lucia Ray, Naseem Dillman-Hasso, Julia F. Strand

**Affiliations:** Department of Psychology, Carleton College, Northfield, Minnesota, United States of America; University of Western Ontario, CANADA

## Abstract

The McGurk effect is a classic audiovisual speech illusion in which discrepant auditory and visual syllables can lead to a fused percept (e.g., an auditory /bɑ/ paired with a visual /gɑ/ often leads to the perception of /dɑ/). The McGurk effect is robust and easily replicated in pooled group data, but there is tremendous variability in the extent to which individual participants are susceptible to it. In some studies, the rate at which individuals report fusion responses ranges from 0% to 100%. Despite its widespread use in the audiovisual speech perception literature, the roots of the wide variability in McGurk susceptibility are largely unknown. This study evaluated whether several perceptual and cognitive traits are related to McGurk susceptibility through correlational analyses and mixed effects modeling. We found that an individual’s susceptibility to the McGurk effect was related to their ability to extract place of articulation information from the visual signal (i.e., a more fine-grained analysis of lipreading ability), but not to scores on tasks measuring attentional control, processing speed, working memory capacity, or auditory perceptual gradiency. These results provide support for the claim that a small amount of the variability in susceptibility to the McGurk effect is attributable to lipreading skill. In contrast, cognitive and perceptual abilities that are commonly used predictors in individual differences studies do not appear to underlie susceptibility to the McGurk effect.

## Introduction

A speaking face provides listeners with both auditory and visual information. Among the most well-documented phenomena in the speech perception literature is the finding that listeners are more successful at understanding speech when they can see and hear the talker, relative to hearing alone [1–5]. Another commonly cited demonstration of the influence of the visual modality on speech perception is the McGurk effect, which occurs when discrepant auditory and visual stimuli result in the perception of a stimulus that was not present in either individual modality [6]. For example, when presented with an auditory /bɑ/ and a visual /gɑ/, participants often report perceiving a fusion of the two syllables, /dɑ/ or /θɑ/. The McGurk effect is a remarkably robust illusion—it occurs when the voice and the face are mismatching genders [7], when the face is represented by a point-light display [8], when listeners are told to focus solely on one modality [9], and when the auditory and visual signals are temporally misaligned [10,11].

Despite the apparent robustness of the McGurk illusion in pooled data, there is substantial variability in the extent to which individual participants are susceptible to the illusion—that is, the rates at which individuals report perceiving fusion responses [12–16]. Although task demands can affect fusion rates (e.g., closed set tasks tend to elicit higher fusion rates than open-set tasks; [14]), substantial variability at the individual level still exists in these studies. In experiments assessing McGurk susceptibility (MGS), some participants consistently perceive fusions when presented with McGurk trials, whereas others rarely or never do, and instead report perceiving the auditory stimulus. This individual variability in MGS is quite striking; fusion rates across individuals can range from 0%–100% [14]. This variability cannot be attributable to measurement error or random noise, as rates of MGS are quite stable within individuals [13], even across delays as long as one year [14].

One consequence of this extreme variability in susceptibility to the McGurk effect is inconsistencies in reported group differences in MGS. For example, Magnotti and Beauchamp [17] recently pointed out that, across studies, group MGS rates of individuals with Autism spectrum disorder were between 45% *lower* and 10% *higher* than control participants. Further, the authors note that the studies with the largest sample sizes have found the smallest group differences in these populations. Thus, it appears that individual differences are the primary contributor to overall variance in MGS, making it particularly difficult to study group differences in MGS [17]; yet, it is still unclear what factors are driving the wide individual variability in MGS.

MGS has classically been assumed to represent individual differences in the ability to integrate auditory and visual information (see [18]), but recent research has cast doubt on this idea. Van Engen and colleagues [19] found no relationship between MGS and visual enhancement—the extent to which an individual’s recognition performance is increased for audiovisual relative to audio-only speech. In addition, McGurk stimuli and congruent speech stimuli differ in the speed with which they are identified [20–23], the cortical regions recruited to process them [24–26], and the subjective ratings of category goodness participants provide for them [27,28]. Debate persists about whether there is a distinct integration stage in which individuals differ even for congruent speech [29], but consensus is building that the ways participants process congruent speech and McGurk stimuli are not equivalent (see [30] for a recent review of these issues).

If individual differences in MGS are not a function of differences in integration skill, then what might be underlying them? One possibility may be that individuals differ in *causal inference*—the extent to which they are able to determine whether the auditory and visual inputs are coming from the same source [31]. Other clues come from work on group-level rates of MGS. These findings suggest that MGS can vary as a function of age [32], gender ([33], but see [14,34]), clinical conditions such as schizophrenia [35] and autism ([36,37] but see [38]), and linguistic or cultural background ([39,40] but see [15]). However, very little work to date has attempted to account for differences in MGS between individuals. Indeed, even when large differences across groups are identified, there remains large variability in MGS within groups (e.g., [36]). Nath and Beauchamp [12] found that greater activity in the left superior temporal sulcus was associated with an increased likelihood that subjects would perceive a McGurk fusion. These results present a compelling case for the involvement of this region in individual differences in MGS, but the roots of these differences and possible behavioral correlates remain an outstanding puzzle in the literature. Here, we identify three classes of explanations (which are not mutually exclusive) for why individuals may differ in MGS.

### Lipreading ability

Given that reporting a fused response relies on combining information from the auditory and visual modalities, differences in MGS may be related to the ability to extract information from the unimodal signals (see [41]). Because normal-hearing participants report fusions even with perfectly intelligible auditory stimuli in the absence of background noise, it is unlikely that individual differences in hearing ability are driving differences in MGS. In contrast, there is considerable individual variability in lipreading ability [42,43], which might be expected to affect MGS rates—individuals who have a reduced capacity to extract meaningful information from the visual signal may be less susceptible to the McGurk effect simply because they are not lipreading well enough to allow visual influence on the auditory signal. To date, there is no evidence that MGS is related to the ability to lipread sentences [44] or consonants [13], but participants who are better able to visually identify the place of articulation (POA) of the consonants do tend to perceive more McGurk fusions [13]. The correlation between this modified measure of lipreading ability and MGS is rather small (*r* = .32), indicating that a large amount of variability in MGS is independent of lipreading ability.

### Phonemic categorization

It may be that individual differences in MGS are not primarily a function of differences in the ability to extract information from the unimodal signals nor differences in the ability to integrate those signals. In fact, it is possible that all listeners (with normal hearing and reasonable lipreading skill) are extracting and integrating the auditory and visual stimuli in a similar way, and the root of individual differences in MGS is how participants assign the audiovisual percept to a category. That is, the wide variability in MGS may have little to do with differences in what people perceive, but may instead reflect differences in how people categorize what they perceive. Brancazio and Miller were the first to suggest that lower MGS may “reflect differences in how the percepts are mapped onto phonetic categories” [45]. Although this hypothesis has not been explicitly tested, there is ample evidence from other areas of research that listeners attend to and encode lower-level phonetic detail during speech perception and therefore have access to more information about a percept than just the phoneme or word they report (e.g., research on the processing of lexically embedded words or effects of talker variability on spoken word recognition [46,47]).

Some work on the McGurk effect has also indicated that the way people categorize McGurk stimuli does not fully describe their perceptual experience with the stimulus. For example, McGurk trials in which participants report perceiving the auditory stimulus may still be influenced by the visual input. Gentilucci and Cattaneo [48] showed that the voice spectra and lip kinematics of participants’ responses to non-fusion responses to McGurk stimuli differed significantly from those to congruent stimuli. This work suggests that although participants did not experience the McGurk effect, they extracted and integrated some information about the visual input which influenced their perception and subsequent production of the auditory stimuli. Similarly, Brancazio and Miller [45] found that visual information from McGurk tokens influenced the perception of voicing, even when participants reported perceiving the auditory stimulus. As a result, the authors argued that the rates of MGS are likely to underestimate the extent to which audiovisual integration has occurred. These results challenge the idea that non-fusion responses reflect failures to extract visual information or a lack of integration.

Further, when listeners experience a McGurk fusion, they may still be sensitive to the fact that the incongruent stimulus is not a perfect exemplar of the perceived phoneme; category goodness ratings are lower for McGurk stimuli resulting in fused percepts than for congruent stimuli [27,28]. In addition, when presented with discrepant audiovisual stimuli, listeners tend to show perceptual adaptation to the auditory component rather than the perceived McGurk token [49–51]. These results complicate the notion that when presented with McGurk stimuli, participants categorically perceive either a McGurk fusion *or* the auditory signal. Thus, the process of labeling a percept is distinct from the perception of it.

Taken together, these findings suggest another possible mechanism that may underlie individual differences in MGS: a listener’s perceptual gradiency versus categoricity. Individuals differ in how categorically phonemic contrasts are perceived; some listeners have more gradient response patterns in how they categorize ambiguous phonemes, whereas others are more categorical [52,53]. Individuals may vary in the flexibility with which they assign a suboptimal token of a specific phoneme (like the /dɑ/ resulting from an auditory /bɑ/ paired with a visual /gɑ/) to a category. Gradient listeners may notice that a McGurk token is a poor exemplar, but given more flexible category boundaries, they are more likely to accept the imperfect token as an acceptable representation of the fusion category. This would suggest that individuals with more flexible phoneme category boundaries—those who perceive auditory speech more gradiently—may be more susceptible to the McGurk effect. In contrast, for categorical perceivers, who require a higher threshold of support to classify a percept as belonging to the category, the imperfect McGurk token is an unacceptable fit for the fusion category, so they instead report the auditory token, which is a near-perfect fit for that category.

### Cognitive abilities

A third possibility is that individual differences in MGS are not specific to speech (e.g., how visual input is extracted, the unimodal signals are integrated, or percepts are assigned to categories), and are instead a consequence of individual differences in lower-level cognitive abilities. One likely candidate for a cognitive predictor of individual differences in MGS is attentional control. Multiple studies have shown that McGurk fusion rates are lower for groups of participants when attention is divided [54–57], suggesting that processing incongruent auditory and visual information requires attentional resources. Thus, individuals with superior attentional control might be expected to show greater MGS because on any given trial, they are less likely to become distracted and devote some of their attentional resources to task-irrelevant demands. In other words, those with superior attentional control are likely to have sufficient attentional resources available to combine the incongruent auditory and visual inputs into a unified percept.

Two other cognitive abilities on which individuals reliably differ are processing speed (PS) and working memory capacity (WMC). To process spoken language, individuals rapidly parse the incoming sensory information and search memory for lexical or phonetic representations that match the input. Thus, the speed and efficiency with which a person can process information and manipulate it in memory robustly affects many measures of language processing. For example, PS is related to speech perception in noise [58], measures of lexical and grammatical development in children [59], text reception threshold [60], reading ability [61], and some measures of listening effort [62]. In addition, WMC is related to susceptibility to the cocktail party phenomenon [63], verbal SAT score [64], reading comprehension [64], absolute pitch learning [65], speech recognition in noise in certain populations [66], and visual attention allocation [67]. Another hint that WMC may modulate individual differences in MGS is the finding that McGurk fusion rates are reduced when participants are asked to complete a simultaneous working memory task [68]. Although the differences were modest, these results suggest that when WMC is taxed, participants are less able to incorporate auditory and visual speech information.

### The current study

The abilities or traits underlying the tremendous variability in MGS remain unknown, and identifying them has the potential to help explain unimodal and multimodal speech processing. In a recent review, Alsius and colleagues [30] noted the importance of understanding why some individuals do not perceive the McGurk effect: “Exploring why these participants process the audiovisual information differently than McGurk perceivers could enormously advance our understanding of the mechanisms at play in audiovisual speech integration.” Thus, the goal of the current study was to evaluate whether individual differences in susceptibility to the McGurk effect relate to other perceptual and cognitive traits. To that end, we used correlational analyses and mixed effects modeling to assess the relationship between MGS and six potential correlates: lipreading ability, ability to extract information about POA from the visual modality, auditory perceptual gradiency, attentional control, PS, and WMC. Data were collected using an online platform (Amazon Mechanical Turk) to help ensure a large and diverse sample.

## Method

Details regarding the pre-registered sample size, exclusion criteria, and analyses can be accessed at osf.io/us2xd. All data, code for analyses, and materials can be accessed at https://osf.io/gz862/.

### Participants

A total of 206 participants were recruited from Amazon Mechanical Turk: 25 in a pilot study designed to ensure that the McGurk stimuli were effective, and 181 in the main experiment—this number of participants was necessary in order to reach our pre-registered sample size of 155 following data exclusion. A power analysis indicated that a sample size of 155 was sufficient to achieve a power of .90 to detect a correlation of *r* = .26—a conservative estimate of the correlation of *r* = .32 between MGS and lipreading POA reported in Strand et al. [13]. The pilot study took approximately 15 minutes and participants were compensated $2.00 for their time, and the main experiment took approximately 30 minutes and participants were compensated $4.50. All procedures were approved by the Carleton College Institutional Review Board, and participants gave their consent electronically.

To meet our pre-registered criterion of 155 participants for the model-building analysis in the main experiment, we collected data from a total of 181 participants. Participants were excluded based on the following pre-registered criteria: poor accuracy on the math portion of the Ospan task (N = 18), slow reaction times on the lexical decision task (N = 3), or slow reaction times on the flanker task (N = 2). Note that we pre-registered that participants who had accuracy levels below 80% on the math portion of the Ospan task would be excluded; this was based on norming done using the Ospan task in our lab with undergraduate students. Our online sample had much lower accuracy at the math task, so the exclusion criterion was relaxed to poorer than chance levels. Data from one participant were lost for the lipreading task due to technical difficulties. In addition, six participants were excluded for poor accuracy at identifying congruent syllables in the McGurk task (given that the auditory syllables were piloted to be highly recognizable, and these stimuli were presented with congruent visual stimuli, accuracy below 90% suggests that participants were not paying attention to the task). We had anticipated that all participants would have near-perfect accuracy at recognizing congruent syllables in the McGurk identification task, so this exclusion criterion was not pre-registered. Decisions that deviated from the pre-registered exclusion criteria were made prior to conducting the main analysis. Because several of the participants met more than one exclusion criterion, a total of 25 participants were excluded from the model building analysis, resulting in 156 participants (one more than our pre-registered criterion).

### General procedure

Participants completed a MGS task and five other tasks that may be expected to predict susceptibility to the McGurk effect: a lipreading task, a visual analogue scale (VAS) task to measure perceptual gradiency, the Eriksen flanker task to measure attentional control, a lexical decision task (LDT) to measure PS, and the operation span (Ospan) task to measure WMC.

Given that we collected data online and the McGurk and VAS tasks require perceiving and responding to auditory stimuli, we wanted to ensure that participants’ devices could play these stimuli and participants were wearing headphones. We employed a recently validated headphone screening designed for conducting auditory research online [69] that participants were required to pass before participating in the experiment. In this task, participants were first asked to set the sound level of their computers to a level that is comfortable when presented with a broad-band speech-shaped noise file. This file was set to be the same amplitude as the speech stimuli used elsewhere in the study. Participants were then presented six trials of three 200 Hz tones and were asked to judge which of the three tones was the quietest. In each trial, one of the three tones was 180 degrees out of phase across stereo channels. The amplitude of this tone is difficult to distinguish from the others over loudspeakers (due to phase cancellation), but sounds much quieter than the other two when wearing headphones. Participants were only allowed to continue to the main study if they responded correctly to five out of the six trials (see [69] for more information).

### Stimuli and individual task procedures

All video stimuli were recorded with a Panasonic AG-AC90 camera, and all auditory stimuli for the McGurk task were recorded at 16-bit, 44100 Hz using a Shure KSM-32 microphone with a plosive screen. Videos were edited with iMovie (version 10.1), ambient noise was removed from audio files with Audacity (version 2.1.2), and audio files were equalized on root-mean-square (RMS) amplitude with Adobe Audition (version 9.2.0). The auditory and visual stimuli were recorded by a female speaker without a noticeable regional accent, with the exception of the VAS stimuli, which were obtained from Kong and Edwards [53]. The experiments were designed and presented via Gorilla (http://gorilla.sc) through the Amazon Mechanical Turk platform.

#### McGurk susceptibility (MGS): Pilot study

Given the wide variability in the extent to which individual stimuli elicit the McGurk effect [14], we conducted a pilot study via Amazon Mechanical Turk to ensure that the incongruent stimuli we created could effectively elicit the McGurk effect. The auditory stimuli had previously been tested in our lab to ensure intelligibility; all tokens included in this experiment were recognized at rates of 95% or higher in an audio-only context. We began by creating eight McGurk tokens for each of seven stimuli that have previously been used in the literature (A_b_V_g_, A_b_V_f_, A_m_V_g_, A_m_V_t_, A_p_V_g_, A_p_V_k_, and A_t_V_b_; [13,15]). Some of the same auditory tokens appeared across McGurk stimuli, but within each McGurk stimulus, the eight unique stimuli contained different auditory and visual tokens. McGurk stimuli were created by aligning the consonant bursts of the two audio tracks, then deleting the unnecessary auditory and visual component. The audio files were shifted if there was any noticeable audiovisual asynchrony. From these seven sets of McGurk stimuli, we selected (via discussions among the authors) four that seemed to be the most likely to elicit fusion responses, then selected the six tokens within each of these four stimuli that were the most compelling to include in the pilot study.

We also planned to include trials with congruent auditory and visual syllables. To ensure that any observed effects could not be attributed to the splicing process, congruent stimuli were created in the same way as the McGurk stimuli by combining two different tokens of the same syllable. The congruent stimuli consisted of the auditory and visual syllables that made up each of the McGurk stimuli and the expected fusions, resulting in eleven congruent stimuli (/bɑ/, /dɑ/, /fɑ/, /gɑ/, /kɑ/, /mɑ/, /nɑ/, /pɑ/, /tɑ/, /θɑ/, /vɑ/). The congruent stimuli were created using the same auditory and visual tokens used for the McGurk stimuli to ensure that the two stimulus types were as similar as possible.

In the pilot study, we presented six tokens of each of four McGurk stimuli (A_b_V_f_, A_b_V_g_, A_m_V_g_, A_p_V_k_) and three tokens of each of eleven congruent stimuli to 25 participants. The congruent trials were included as fillers out of concern that prolonged exposure to incongruent speech might reduce McGurk fusion rates (see [23,70]), and to ensure that if participants were not susceptible to the McGurk effect, they did not stop attending to the visual modality. However, only McGurk trials were included in the primary analyses. Each McGurk token was presented three times, and each congruent token was presented twice, for a total of 72 McGurk and 66 congruent randomly intermixed trials.

Following presentation of each syllable, a text box appeared on the screen, and participants typed the syllable they perceived. Stimulus presentation was pseudorandomized, and the interstimulus interval was 750 ms. Following the recommendations of Basu Mallick et al. ([14]; see also [15,19]), both /dɑ/ and /θɑ/ were scored as fusion responses for A_b_V_g_ stimuli, and both /tɑ/ and /θɑ/ were scored as fusion responses for A_p_V_k_ stimuli. Consistent with prior research (e.g. [13,14]), we observed wide variability in MGS across participants (mean: 44.9%; SD: 26.4%; range: 0% to 98.6%) and tokens (mean: 44.9%; SD: 13.2%; range: 6.7% to 68%), confirming that the stimuli we used could successfully elicit the McGurk effect.

#### McGurk susceptibility (MGS): Main experiment

The main experiment included each of the 24 McGurk tokens from the pilot study. Table 1 shows the four McGurk stimuli we used in this experiment and expected fusions. The stimuli and procedures in the MGS task were identical to those in the pilot study, and the stimuli were repeated the same number of times.

**Table 1:** McGurk stimuli and expected fusions.

Auditory Stimuli	Visual Stimuli	Expected Fusions
bɑ	gɑ	dɑ, ðɑ, θɑ
bɑ	fɑ	vɑ
mɑ	gɑ	nɑ
pɑ	kɑ	tɑ, ðɑ, θɑ

#### Lipreading ability

Lipreading ability was measured using a visual-only consonant recognition task based on that employed by Strand et al. [13]. Including this task allowed us to attempt to replicate the finding that lipreading ability is related to MGS [13], and determine the extent to which various cognitive abilities are related to MGS after controlling for lipreading ability. Lipreading stimuli consisted of three tokens of each of ten syllables: /bɑ/, /dɑ/, /fɑ/, /gɑ/, /kɑ/, /mɑ/, /nɑ/, /pɑ/, /tɑ/, /vɑ/. Each token was presented twice, resulting in 60 lipreading trials (10 syllables * 3 tokens * 2 repetitions), with an interstimulus interval of 750 ms. Participants responded by typing what they perceived into a text box. Lipreading ability was quantified by-participants as the proportion of correct responses. Stimulus presentation order was pseudorandomized, and participants completed four practice trials before beginning.

We opted to use consonants rather than words to make the McGurk and lipreading tasks as similar as possible, and to enable us to measure the ability to accurately lipread POA, which provides a more fine-grained analysis of participants’ lipreading abilities because POA is the most readily available feature of the visual signal [13,71,72]. Following the convention of Strand et al. [13], we used the following consonant groupings for POA: bilabial (b, p, m), labiodental (f, v), velar (k, g), and alveolar (d, l, n, s, t, z). Given that POA recognition is a more sensitive measure of lipreading ability than consonant recognition, and lipreading POA has been shown to correlate with MGS [13], we included lipreading POA rather than raw lipreading score in the model building analysis.

#### VAS rating task

Perceptual gradiency was measured using a continuous VAS task, which has been shown to be sensitive to individual differences in phoneme categorization [53,73–78], and is less susceptible to task-related biases than categorical judgments [76]. In VAS tasks, participants are provided with a line with endpoints representing the extremes of a continuum, like /s/ on the left end and /∫/ on the right end of a centroid frequency continuum [73]. Participants are then presented with stimuli that vary continuously on some dimension (like centroid frequency or voice onset time), and are asked to click on the line where they believe the stimulus falls (e.g., between /s/ and /∫/). Some individuals respond rather categorically, with most responses clustered on the extremes of the continuum, and others respond more gradiently, with responses distributed throughout the continuum. VAS ratings have been shown to be correlated with true acoustic parameters of the stimulus [73,78,79], a finding that runs counter to the claims of traditional categorical perception experiments [80] and suggests that listeners are indeed sensitive to within-category covert contrasts. Julien and Munson [73], showed that as the centroid frequency changed from more like /s/ to more like /∫/, participants were more likely to rate the token as /∫/. This indicates that certain listeners were actually more sensitive than other listeners to these phonetic differences that were present in the stimuli, and were not just more willing to respond gradiently regardless of the input. Furthermore, this measure has been shown to be reliable—participants are consistent in their manner of responding across test days [53].

Stimuli for the VAS task consisted of a /dɑ/ to /tɑ/ continuum varying in both voice onset time (VOT) and fundamental frequency (f_0_). We considered using several different continua, but since much of the existing research using the VAS task to measure perceptual gradiency relies on a single continuum [53,76,78], we opted to only use the /dɑ/ to /tɑ/ continuum. Stimuli were obtained from Kong and Edwards [53], and consisted of six log-scale VOT steps, and at each VOT step there were five f_0_ steps (for more information about stimulus creation, see [53]). Following the procedures of Kong and Edwards [53], the 30 stimuli (6 VOT steps * 5 f_0_ steps) were presented three times, for a total of 90 VAS trials. After the presentation of each syllable, a line with a slider at the midpoint appeared on the screen, and participants were asked to click on (or move the slider to) the location on the continuum where they believed the stimulus fell. The voiced consonant (/dɑ/) always appeared on the left end of the line, and the unvoiced consonant (/tɑ/) always appeared on the right end of the line. To be consistent with prior research, the VAS line was unlabeled, but the values ranged from 0 (/dɑ/) to 535 (/tɑ/), with a midpoint of 268 [76,78]. Participants were encouraged to use the entire line if they felt it was appropriate [75,76,78]. Stimulus presentation was pseudorandomized, and the interstimulus interval was 350 ms.

To quantify the extent to which a participant perceived the VAS stimuli gradiently or categorically, we fit a polynomial function to each participant’s VAS data and used the coefficient of the quadratic term as a measure of gradiency (following the procedures of [53]). A small coefficient indicates more gradient perception, and a large coefficient indicates a more categorical response pattern.

#### Attentional control

Attentional control was measured using the Eriksen flanker task [81] with arrows rather than letters [82]. Participants were presented with a row of five arrows pointing either to the left (“<”) or to the right (“>”), and were asked to press the /e/ key if the central arrow pointed to the left, and the /i/ key if the central arrow pointed to the right. Participants were asked to respond as quickly and accurately as possible. On congruent trials, the flanker arrows pointed in the same direction as the target arrow (e.g. < < < < <), and on incongruent trials, the flanker arrows pointed in the opposite direction as the target arrow (e.g., > > < > >). Reaction times to incongruent trials tend to be slower than those to congruent trials, indicating an attentional cost for resolving the incongruity [81–83]. Thus, the average difference in reaction time for correct responses between congruent and incongruent trials was used as a measure of inhibitory control, such that higher values indicate worse inhibition.

After eight practice trials with feedback, participants completed a total of 90 trials in a pseudorandomized order (45 congruent and 45 incongruent, intermixed). During each trial, the flanker and target arrows appeared on the screen simultaneously, and the interstimulus interval was 750 ms. We ensured that there were no repeated identical targets throughout the task (e.g., < < > < < was never followed by < < > < <) in an attempt to minimize any sequential trial effects (see [82–85]), even though stimulus presentation order was consistent across participants.

#### Processing speed (PS)

PS was measured with a standard lexical decision task (LDT [86]). Participants were presented with four-letter strings (e.g., “BORN” or “BILK”), and were asked to determine as quickly and accurately as possible whether the string formed a real English word, and indicate their response by pressing either “e” (for “yes,” it is an English word) or “i” (for “no,” it is not an English word) on a keyboard. We used these letters rather than “y” and “n” to ensure that participants used two hands to complete the task. All words were common (SUBTLEX-US log frequencies above 3), and nonwords were phonotactically legal one-letter substitutions of real English words. After completing five practice trials with feedback (three words and two nonwords), participants completed 80 experimental trials (40 words and 40 nonwords) in a pseudorandomized order, with an interstimulus interval of 750 ms. Processing speed was determined by calculating the average reaction time to correct responses.

#### Working memory capacity (WMC)

WMC was evaluated with a standard operation span (Ospan) task [87,88]. Participants were presented with a series of interleaved simple math problems and unrelated words, and were asked to verify whether the equation was true while they attempted to remember the list (e.g., “(7–3) x 3 = 12” followed by presentation of the word “farm”). Participants’ responses (either “t” for true or “f” for false) prompted a 500 ms delay followed by presentation of the word, and the word remained on the screen for 1000 ms. If participants did not respond to the math equation within 5000 ms, the word appeared on the screen, and the trial was scored as “no response” for the math equation. There was a 1000 ms interstimulus interval between the word and the next equation. Half of the equations were correct, and half were incorrect.

Although each set size is typically presented three times [87,88], we opted to shorten the task by removing one set each of sizes two, three, and five (following the recommendation of [89]). Thus, participants in this task completed two sets of size two, two sets of size three, three sets of size four, two sets of size five, and three sets of size six, for a total of 50 equation/word pairs across 12 sets. After each set, participants were prompted with a text box to type the words in the order they were presented, one at a time. Set size and presentation of stimuli within each set was pseudorandomized, and no equation or word appeared more than once. Participants who performed below 50% on the math problems were excluded from analysis. This step was taken to ensure that participants did not trade off math and recall accuracy, or ignore the math problems altogether. Prior to beginning the task, participants completed two practice trials, one of set size two and one of size three. The task was scored by summing the sizes of all sets in which each item was recalled correctly in order, resulting in a score ranging from 0 to 50.

## Results

We first calculated descriptive statistics for each of the tasks to ensure that the values we obtained had good variability and reasonable means [13] (i.e., comparable to those reported elsewhere in the literature; see, for example, [14,62,90]). Descriptive statistics for the six tasks (seven sets of values including both measures of lipreading ability) can be found in Table 2.

**Table 2:** Summary statistics for all tasks.

Task	N	Mean (SD)	Range
MGS	175	0.54 (0.29)	0–0.99
Lipreading	180	0.32 (0.07)	0.08–0.60
Lipreading POA	180	0.75 (0.09)	0.18–0.92
VAS	181	0.40 (0.58)	-0.73–1.69
Flanker	179	36 (30)	-17–158
LDT	178	632 (82)	484–871
Ospan	163	21.24 (10.92)	0–50

Note. MGS is measured in proportion of responses to incongruent stimuli that were scored as fusion responses. Lipreading scores are measured in proportion correct. VAS scores are scaled for ease of interpretation. Flanker and LDT are measured in reaction time (RT). Ospan is measured on a scale from 0 through 50. RTs are in milliseconds. MGS = McGurk susceptibility; POA = place of articulation; VAS = visual analogue scale score; Flanker = Flanker test (mean incongruent RT—mean congruent RT); LDT = lexical decision task.

**Fig 1:**
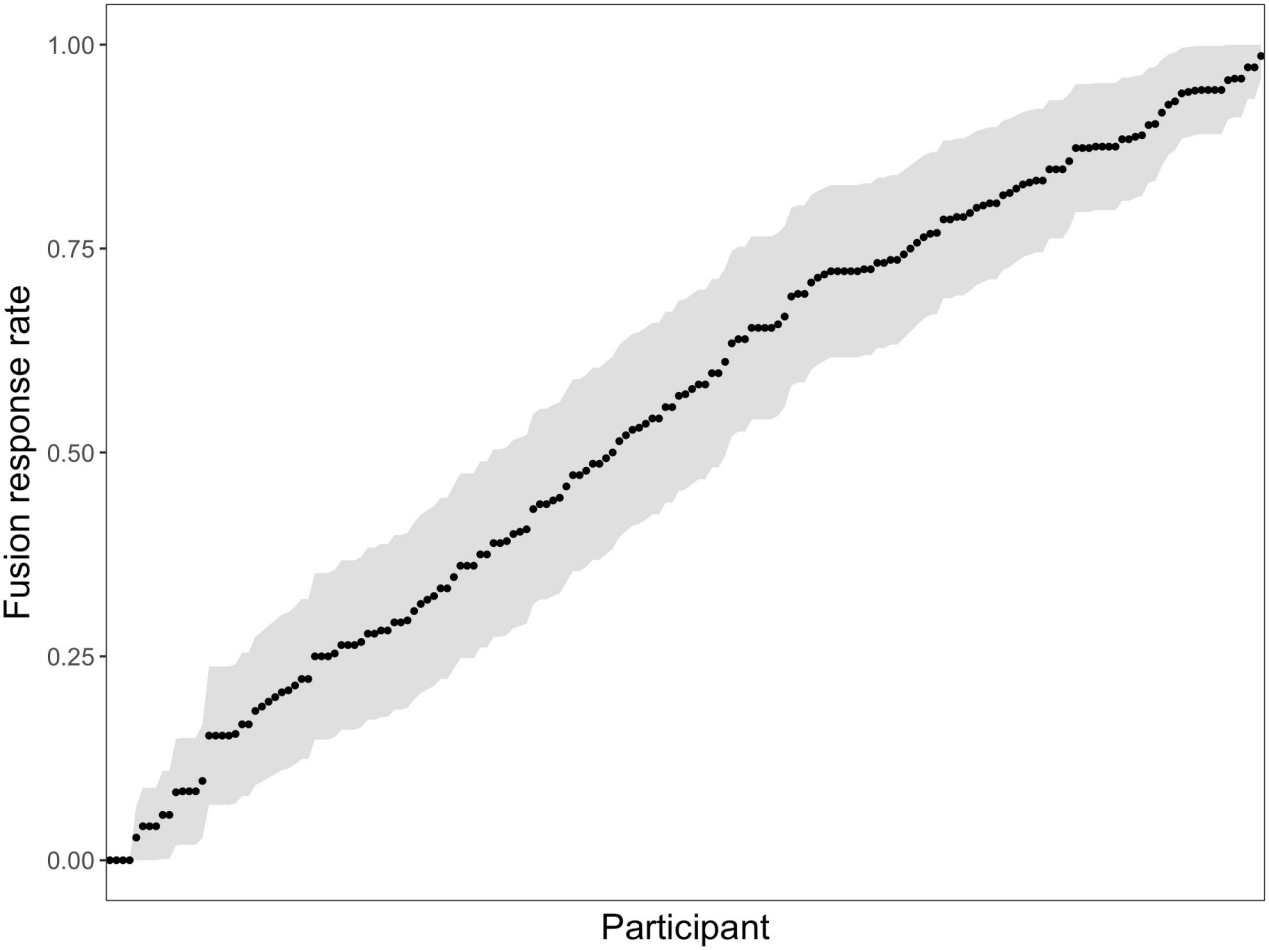
Mean by-participant McGurk fusion rate in ascending order. Shaded region represents two standard errors from each participant’s mean fusion rate. N = 175.

The descriptive statistics for each of the tasks are comparable to those reported previously. MGS ranged from a 0% fusion rate (i.e., a participant never reported a fused percept) to a 99% fusion rate (Fig 1), suggesting that our measure of MGS accurately captured the range of values that have been reported in numerous other experiments.

**Fig 2:**
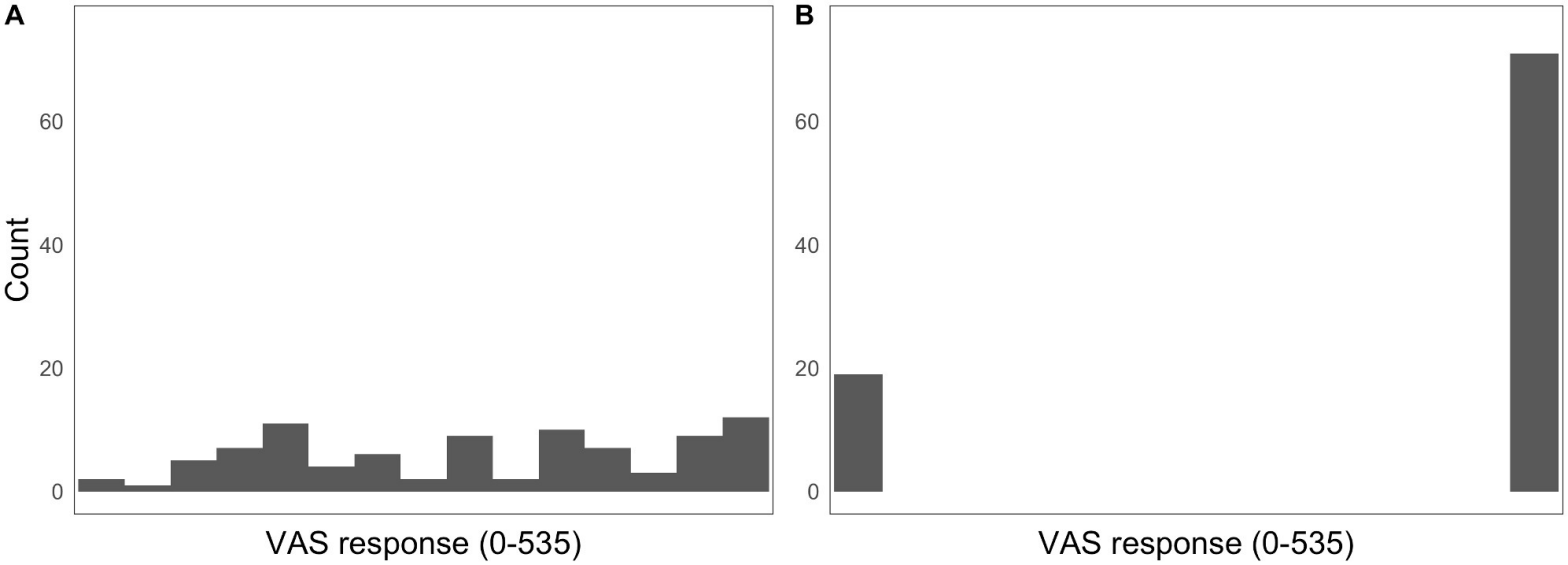
Distribution of VAS responses for a representative gradient (A) and categorical (B) listener. VAS = visual analogue scale.

The means and ranges for both lipreading tasks were comparable to those reported in Strand et al. [13], and click distributions for the VAS task represented a wide range from highly categorical listeners (Fig 2A) to gradient listeners (Fig 2B). The results from the three cognitive measures were also consistent with what has been shown previously. Responses to incongruent stimuli in the flanker task tended to be slower than those to congruent stimuli [81], and the mean and standard deviation in the LDT were very similar to those reported in Strand et al. [62]. Finally, though the mean and range of Ospan scores were larger than has been reported previously (current study: mean = 21.24, range = 0–50; [88]: mean = 11.43, range = 0–38), scores covered the full range of possible values and the distribution of scores approximates a normal distribution.

**Fig 3:**
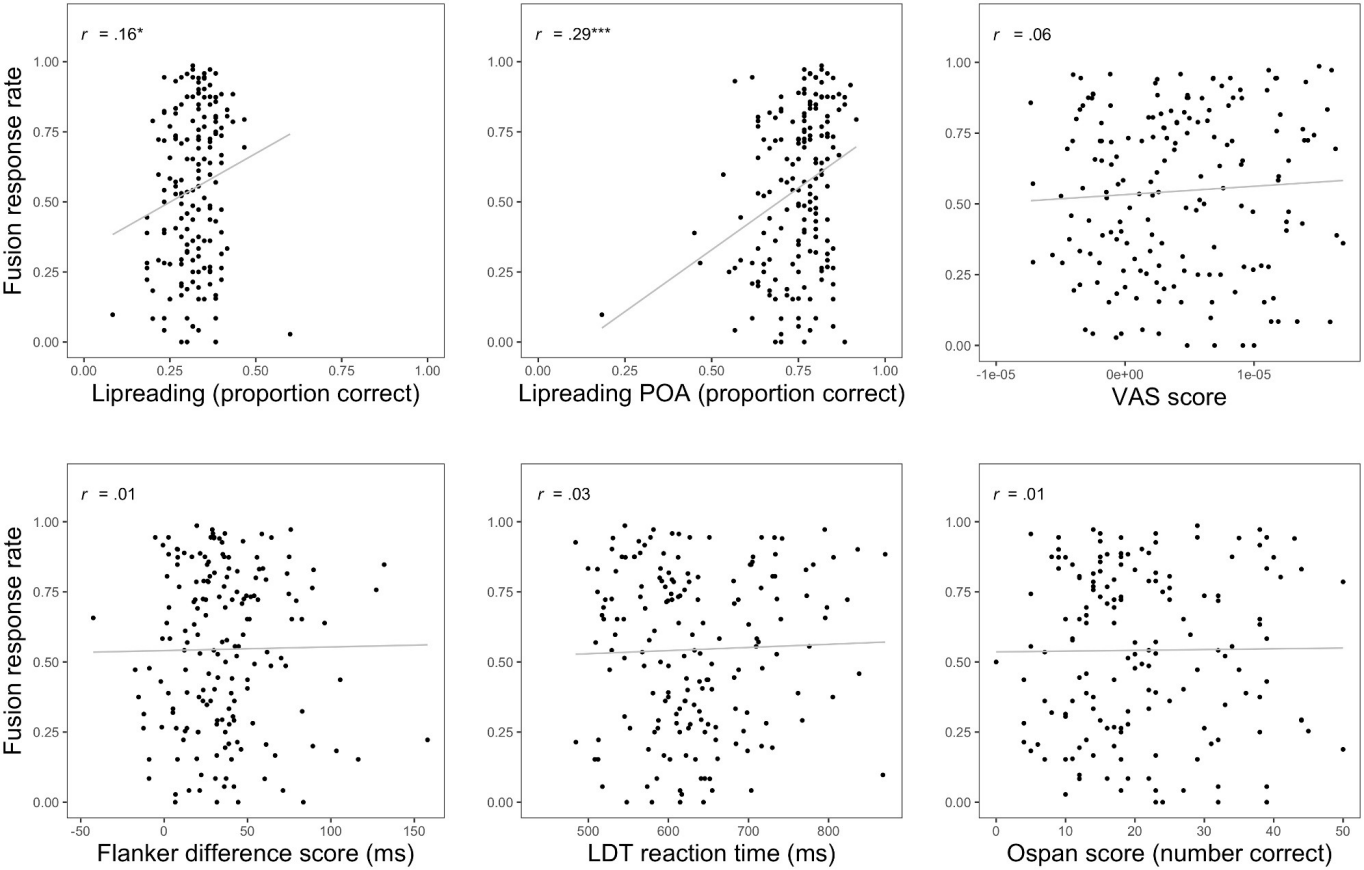
Scatterplot and correlations (*r* values; *** *p* < .001; * *p* < .05) showing the relationship between MGS and each of the predictor variables: Lipreading, lipreading place of articulation (POA), perceptual gradiency (visual analogue scale task; VAS), attentional control (flanker), processing speed (lexical decision task; LDT), working memory capacity (operation span; Ospan). Line represents regression line of best fit. Raw VAS scores are shown here whereas centered and scaled scores are shown in Table 2 for ease of interpretation. Note that one participant had a particularly low lipreading POA score and also had a relatively low MGS fusion rate (top row, middle panel). To ensure that this participant’s data were not driving the observed correlation between fusion rate and POA score, we performed an exploratory analysis computing this correlation without that single participant. Results were very similar to those reported in the text (*r* = 0.27; *p* < .001).

We conducted two sets of analyses to determine the extent to which each of the cognitive and perceptual traits were related to susceptibility to the McGurk effect. In the first set of analyses, we conducted Pearson correlations between MGS and each of the predictors, including both methods of scoring lipreading ability. Because six participants were eliminated from the MGS task, and different numbers of participants were eliminated from each of the remaining tasks, the sample sizes in the correlational analyses ranged from 160 to 175. The only correlations that emerged significant were between MGS and lipreading ability, both raw scores and POA (*r* = .16 and *r* = .29, respectively; see Fig 3). However, as can be seen in Fig 3, the predictive validity of POA is quite low (root-mean-square error = .27; mean-square error = .07).

In the next set of analyses, data were analyzed using linear mixed effects models via the *lme4* package in R (version 3.4.0; [91]). We first built a full model containing each of the five centered and scaled predictors, using lipreading POA as a measure of lipreading ability. We included lipreading POA rather than raw lipreading score given its stronger correlation with MGS. We then selectively removed variables based on significance and contribution to the total sum of squares, and compared models using likelihood ratio tests via the *lmerTest* package [92]. All mixed effects models utilized the maximal random effects structure justified by the design [93]. Likelihood ratio tests indicated that a model containing lipreading POA provided a better fit for the data than an intercept-only model (χ^2^_1_ = 11.49, *p* < .001), and that a model with all predictors was not a better fit than a model with only lipreading POA (χ^2^_4_ = 2.26; *p* = .69).

The model including only lipreading ability also had the lowest Akaike Information Criterion (AIC) and Bayesian information criterion (BIC) of all models we built (see Table 3), indicating that it provided a better fit for the data than the other models. AIC and BIC are model selection criteria based on maximum likelihood estimation, and though both include a penalty term for the number of parameters in the model (i.e., they penalize overfitting), the penalty term is more severe in the BIC. The summary output from the model including lipreading POA as the only predictor of MGS indicated that individuals with better lipreading POA ability were more susceptible to the McGurk effect (β = .48, *SE* = .14, *z* = 3.46, *p* < .001; recall that the lipreading measure is represented in standardized units). Thus, consistent with the correlational analyses, the model-building analysis indicated that only lipreading ability (as measured by the lipreading POA measure) was related to MGS, though it is worth noting that, in line with the results of Strand et al. [13], the effect of including information about lipreading POA was relatively modest.

**Table 3:** Akaike Information Criterion (AIC) and Bayesian information criterion (BIC) for each of the mixed effects models compared. AIC and BIC values shown here are relative to the intercept-only model. Therefore, negative numbers indicate that a model is better fit for the data than the intercept-only model.

Model	AIC	BIC
Flanker + LDT + Ospan + VAS + lipreading POA	-3.75	32.79
LDT + VAS + lipreading POA	-7.58	14.35
Lipreading POA	**-9.49**	**-2.18**

The literature on the McGurk effect is inconsistent as to which types of responses to McGurk stimuli are scored as fusion responses. In our primary analysis, we used a scoring method in which the precise fusion responses are strictly defined (see Table 1), as this is a widely used scoring method [7,12–15]. However, some researchers have argued that this method is too stringent (see [26,30,94]), and instead advocate quantifying MGS as rates at which participants report anything other than the auditory stimulus. Thus, we performed an additional exploratory analysis in which we conducted the correlational and model-building analyses described above using this more flexible scoring method. This analysis yielded a very similar pattern of results; indeed, MGS rates derived from our original scoring method and the more flexible method were almost perfectly correlated (*r* = .96, *p* < .001).

## Discussion

The McGurk effect is a robust illusion for which no cognitive or perceptual correlates have been identified in the published literature. This experiment served as the first large-scale correlational study of the relationship between MGS and multiple cognitive and perceptual abilities that are prevalent in the speech perception literature. Using both a correlational analysis and mixed effects modeling, we found no evidence that perceptual gradiency, attentional control, PS, and WMC predict individual differences in MGS. However, we found that participants who were better able to lipread consonants and extract POA information from the visual modality were more susceptible to the McGurk effect. The lipreading results are in agreement with those observed in Strand et al. [13]; indeed, the magnitudes of the correlations between MGS and lipreading POA were quite similar in the two studies (*r* = .32 in Strand et al. [13]; *r* = .29 in the current study), despite one being conducted in a laboratory setting and the other being conducted online. Similarly, the magnitudes of the correlations were nearly identical when lipreading ability was measured using the standard scoring method (*r* = .14 in Strand et al. [13]; *r* = .16 in the current study). It is worth noting that the root-mean-square error of a model predicting MGS from lipreading POA was rather high (0.27), suggesting that although the relationship between MGS and lipreading POA is reliable, having an individual’s lipreading POA score does not allow for very accurate prediction of their susceptibility to the McGurk effect.

We had hypothesized that individuals who perceive auditory speech more gradiently, and thus have more flexible phoneme categories, would be more susceptible to the McGurk effect because they would be more willing to assign an imperfect McGurk token to the fusion category. Correspondingly, we predicted that when categorical perceivers with strict phoneme category boundaries encountered an imperfect McGurk token that was an unacceptable fit for the category representing the fused percept, they would instead report the auditory token (which a pilot study determined to be a highly recognizable token of that syllable). Contrary to our hypothesis, results showed no evidence that perceptual sensitivity to ambiguous phonemes was related to MGS. A limitation to using the VAS task is that it is a measure of auditory gradiency; future research should attempt to evaluate whether performance on tasks of audiovisual gradiency may predict MGS.

Dividing attention reduces McGurk fusion rates [54–57], so we had expected that individuals with greater attentional control, who are better able to inhibit distracting information and are therefore less prone to dividing their attention during the task, would be more susceptible to the McGurk effect. Similarly, engaging in a concurrent working memory task reduces rates of MGS [68], so we had hypothesized that individuals with greater WMC would have higher MGS. Thus, the observed lack of relationship between MGS and both attentional control and WMC is somewhat surprising. It is conceivable that the relationship would have emerged if we had used a different measure of attentional control—like the Simon task [95] or the Stroop task [96]—but in the absence of a clear prediction about why these tasks would be expected to have different relationships with MGS, this explanation is unlikely. Rather, it appears that an individual’s ability to inhibit irrelevant stimuli is unrelated to their susceptibility to the McGurk effect. Another possible explanation for the lack of relationship between MGS and attentional control is that although the results from the flanker task were comparable to those reported in other studies, this task has relatively low between-participant variability, making it difficult for significant correlations to emerge—indeed, difference scores tend to have lower between-participant variances than the component values from which they are derived [97].

Although cognitive research is most commonly conducted in laboratory settings, researchers are increasingly using online venues to collect data. Validation studies have attempted to evaluate whether and how data collected online differs from laboratory data [83,98,99]. The largest such cognitive study to date indicated that a range of reaction time tasks, including the Stroop and Simon tasks, task-switching, and a flanker task similar to the one used in this study, were replicated in online samples [98]. Fewer speech perception studies have been conducted online (e.g., [14]), but the existing research has also shown consistency in in-lab and online data. For example, Slote and Strand [100] showed correlations among word recognition accuracy data collected on Amazon Mechanical Turk and in the lab of *r* = .87, and correlations among auditory LDT latencies from both sources of *r* = .86, suggesting strong similarities between online and in-lab data. In addition, the component of the study that was an attempted replication (the relationship between MGS, lipreading, and lipreading POA) rendered results that were very similar to what had been reported previously using an in-lab sample [13].

One concern about online data collection that is particularly relevant to audiovisual speech experiments is that poor auditory or visual quality may cloud effects that would be observable in a laboratory setting (but see [14,101] for other examples of online studies on the McGurk effect). To address this issue, we ensured that participants had sufficiently good auditory equipment by employing a recently introduced headphone screening [69]. Although we did not control for video quality, if video quality was poor, we would expect to observe lower fusion rates because visual degradation tends to reduce McGurk fusion rates [40,102–104]. However, the McGurk fusion rates we observed were comparable to what has been reported previously, and covered the full range from 0% to 99%; in fact, fusion rates in our study were slightly higher than those reported elsewhere [6,13–15], which is likely attributable to the fact that a pilot study helped identify effective McGurk tokens, and suggests that video quality was not a crucial issue. Thus, the null effects observed here are not likely to be a function of the fact that the study was conducted online. A final concern about online measures of individual differences in reaction time is that differences in participants’ hardware or software have the potential to confound individual differences in processing speed. Note, though, that this is less of a concern for the flanker task, given that scores from it are difference scores (timing for incongruent minus congruent stimuli) rather than absolute reaction times.

At the time of writing this paper, the original McGurk study had been cited over 6,000 times and has had a tremendous influence on audiovisual speech research (see [105]). Given the importance of the paradigm in the literature, it is quite surprising that the factors influencing the large and well-documented individual variability in MGS have not received more attention (but see [12]). However, it is possible that other research teams have attempted the same type of study presented here, but the prevalence of publication bias [106–109] rendered studies that failed to find a relationship between MGS and perceptual or cognitive traits too difficult to publish, exacerbating the file drawer problem [106] and making these results inaccessible to other researchers. Thus, these null effects reported here may be particularly informative to other research teams who are seeking to identify correlates of individual differences in MGS—indeed, the three cognitive abilities we included are commonly used predictors in individual differences studies. What, then, is driving the substantial variability in MGS? Perceptual and cognitive correlates of susceptibility to the McGurk effect remain elusive, and future research should aim to identify other sources of variability in MGS.
